# Inhibition of autophagy-attenuated calcium oxalate crystal-induced renal tubular epithelial cell injury *in vivo* and *in vitro*

**DOI:** 10.18632/oncotarget.23383

**Published:** 2017-12-17

**Authors:** Yunlong Liu, Derong Li, Ziqi He, Quan Liu, Jihua Wu, Xiaofeng Guan, Zhiwei Tao, Yaoliang Deng

**Affiliations:** ^1^ Department of Urology, The First Affiliated Hospital of Guangxi Medical University, Nanning, China

**Keywords:** autophagy, calcium oxalate crystals, renal tubular epithelial cells, reactive oxygen species, autophagic vacuoles

## Abstract

Accumulating evidence suggests that autophagy is involved in the pathophysiological processes of kidney diseases. However, the role of autophagy in the formation of calcium oxalate (CaOx) nephrolithiasis remains unclear. In this study, we investigated the effects of autophagy on renal tubular epithelial cell injury induced by CaOx crystals *in vivo* and *in vitro*. We first observed that the expression levels of LC3-II and BECN1 and number of autophagic vacuoles were markedly increased in the renal tissue of CaOx stone patients. We subsequently found that exposure of HK-2 cells to CaOx crystals could increase LC3-II and BECN1 expression as well as the number of GFP-LC3 dots and autophagic vacuoles in a dose- and time-dependent manner. In addition, our results suggest that CaOx crystals induced autophagy, at least in part, via activation of the reactive oxygen species (ROS) pathway in HK-2 cells. Furthermore, inhibition of autophagy using 3-methyladenine or siRNA knockdown of BECN1 attenuated CaOx crystal-induced HK-2 cells injury. However, enhancing autophagic activity with rapamycin exerted an opposite effect. Taken together, our results demonstrate that autophagy is essential for CaOx crystal-induced renal tubular epithelial cell injury and that inhibition of autophagy could be a novel therapeutic strategy for CaOx nephrolithiasis.

## INTRODUCTION

Nephrolithiasis has a serious impact on human health and is a frequently occurring disease that has many causes, including genetic, environmental, and metabolic factors. However, its pathogenesis is still not fully understood [1]. The formation of kidney stones includes urinary supersaturation, calcium salt crystal adhesion, aggregation, nucleation and growth processes [2]. Common kidney crystals mainly include calcium oxalate (CaOx) crystals, calcium phosphate (CaP) crystals and uric acid salts, of which CaOx crystals are the main crystalline composition of kidney stones [3]. During the formation of stones, renal tubular epithelial cells are exposed to these crystals. Previous studies have shown that exposure of renal tubular epithelial cells (RTECs) to high concentrations of CaOx crystals leads to the production of reactive oxygen species (ROS) and oxygen free radicals and the development of oxidative stress (OS) along with injury and inflammation [4, 5]. Therefore, these results indicate that peroxidatic injury of renal tubular epithelial cells caused by ROS may contribute to the development and progression of calcium oxalate kidney stones.

A growing number of studies have demonstrated that ROS production drives autophagy activation [6–8]. Autophagy is a phenomenon that exists widely in eukaryotic cells and is responsible for the degradation of damaged organelles and long-lived proteins, providing the necessary materials for the synthesis and renewal of proteins and organelles, as well as maintaining cellular metabolism and homeostasis [9, 10]. Autophagy is a dynamic process that mainly involves sequestration of nonessential intracellular organelles and proteins in a double-membrane vesicle called the autophagosome, followed by fusion with lysosomes to form the autolysosome, resulting in degradation of the enclosed materials [11]. MAP1LC3/LC3 (microtubule-associated protein 1 light chain 3), BECN1/Atg6, and Atg5 are the most widely used proteins for monitoring the formation of autophagosomes [12].

Recently, evidence has shown that autophagy is associated with renal diseases [13]. ROS-mediated autophagy has been reported to protect renal tubular epithelial cells from injury induced by urinary proteins [14]. However, Xu and colleagues demonstrated that TGF-β1 activated autophagy through the generation of ROS, which exacerbates TGF-β1-mediated apoptosis in renal tubular epithelial cells [15]. These data suggest that autophagy activation can play a pro-survival or pro-death role under different pathological conditions. However, the role of ROS-mediated autophagy on renal tubular epithelial cells remains unclear in the context of calcium oxalate kidney stones. In the present study, we investigated whether CaOx crystal-mediated ROS could activate autophagy in renal tubular epithelial cells and then examined the role of autophagy in CaOx crystal-induced cell injury.

## RESULTS

### Autophagic activity is increased in the kidney of calcium oxalate nephrolithiasis patients

**Figure 1:**
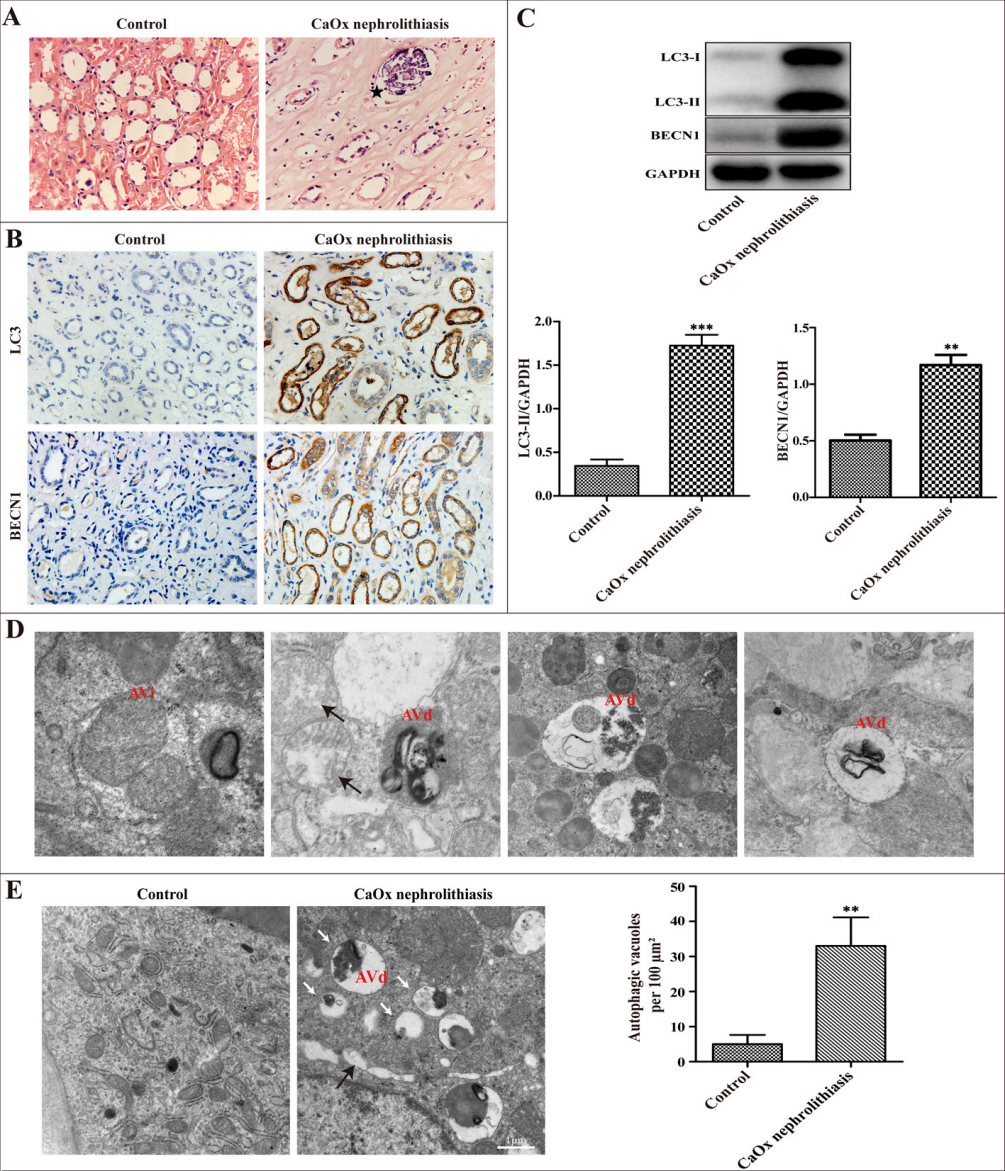
Autophagic activity is increased in the kidney of CaOx nephrolithiasis patients (**A**) Representative images of H&E staining showed crystal deposition in the lumens of the renal tubules of CaOx nephrolithiasis patients (black asterisk); scale bar: 50 μm. (**B**) Immunohistochemical analysis of LC3 and BECN1 expression in CaOx nephrolithiasis tissues and the controls; scale bar: 50 μm. (**C**) A representative immunoblot and quantification analysis of LC3-II and BECN1 in CaOx nephrolithiasis tissues and the controls. (**D** and **E**) Representative transmission electronic micrographs showing autophagic vacuoles in CaOx nephrolithiasis patients and the controls. TEM images showed a typical initial autophagic vacuoles (AVi) and late/degradative autophagic vacuoles (AVd). Mitochondria were swollen and damaged in CaOx nephrolithiasis patients (black arrows) (D); scale bar: 500 nm. The number of autophagic vacuoles per 100 μm^2^ was determined in transmission electron micrographs. White arrows indicated autophagic vacuoles (E); scale bar: 1 μm. Data are presented as the mean ± SD from three experiments. ^**^P < 0.01, ^***^P < 0.001 versus the control group.

Compared with controls, we found CaOx crystal deposition in the lumens of the renal tubules of nephrolithiasis patients examined by H&E staining (Figure 1A). To test whether autophagy activity is increased in calcium oxalate nephrolithiasis tissues, the expression of critical autophagic proteins LC3 and BECN1 was evaluated by immunohistochemistry and western blot technology. We observed that the expression levels of LC3-II and BECN1 were significantly increased compared to the normal kidney tissues (Figure 1B–1C). To further confirm the induction of autophagy in the kidney of CaOx nephrolithiasis patients, the presence of autophagic vacuoles was detected in kidney tissues using transmission electron microscopy. Autophagosomes, also called initial autophagic vacuoles (AVi), typically have a double membrane, which contain intact cytoplasmic material and/or organelles [11]. Late/degradative autophagic vacuoles/autolysosomes (AVd) typically have only one limiting membrane, which contain cytoplasmic constituents at various stages of degradation [16–18]. As shown in Figure 1D, our present results revealed the dynamic process of a typical AVi or AVd. Compared with the control group, the number of autophagic vacuoles was markedly increased in the renal tissue of CaOx nephrolithiasis patients (Figure 1E).

### Autophagy was induced in HK-2 cells in response to CaOx crystal treatment

**Figure 2:**
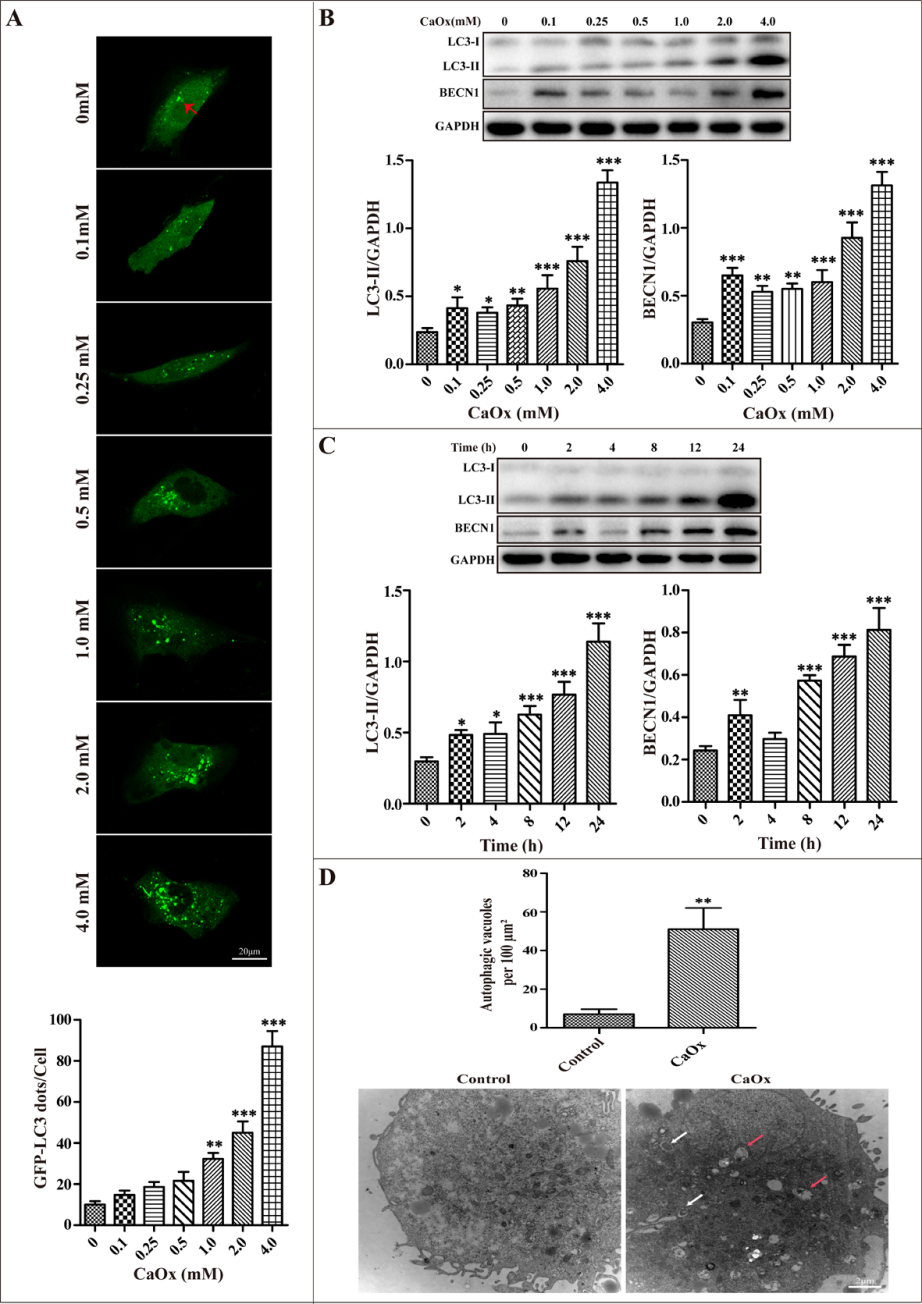
CaOx crystals induced autophagy in HK-2 cells (**A**) The formation of GFP-LC3 puncta was analyzed using confocal microscopy after exposure to different concentrations CaOx crystals for 24 h (red arrows). GFP-LC3 dots/cell were quantified; scale bar: 20 μm. (**B**) A representative immunoblot and quantification analysis of LC3-II and BECN1 as assayed after exposure to different concentrations of CaOx crystals for 24 h. GAPDH was used as a loading control. (**C**) A representative immunoblot and quantification analysis of LC3-II and BECN1 as assayed after exposure to 4 mM CaOx crystals at various time points. (**D**) Representative transmission electronic micrographs showed a markedly increased number of autophagic vacuoles after treatment with vehicle or CaOx crystals (4 mM) for 24 h. The number of autophagic vacuoles per 100 μm^2^ was determined in transmission electron micrographs. White and red arrows indicated autophagosomes and autolysosomes, respectively; scale bar: 2 μm. Data are presented as the mean ± SD from three experiments. ^*^P < 0.05, ^**^P < 0.01 and ^***^P < 0.001 versus the control group.

HK-2 cells were transiently transfected with GFP-LC3 plasmid and then treated with 0 to 4 mM of CaOx crystals for 24 h, followed by examination by confocal microscopy. Figure 2A showed that the number of GFP-LC3 dots gradually increased in a dose-dependent manner. Western blotting analysis also demonstrated a dose-dependent induction of LC3-II and BECN1 by CaOx crystals in HK-2 cells (Figure 2B). Meanwhile, a time-dependent increase of LC3-II formation was demonstrated when cells were treated with of CaOx crystals (4 mM), and the peak of the increase was observed at 24 h post-treatment (Figure 2C). By transmission electronic microscopy (TEM), we further clarify that autophagic vacuoles significantly increased in cells after exposure to CaOx crystals (4 mM) for 24 h compared with the controls (Figure 2D).

### CaOx crystals induced autophagy via activation of the ROS pathway

**Figure 3:**
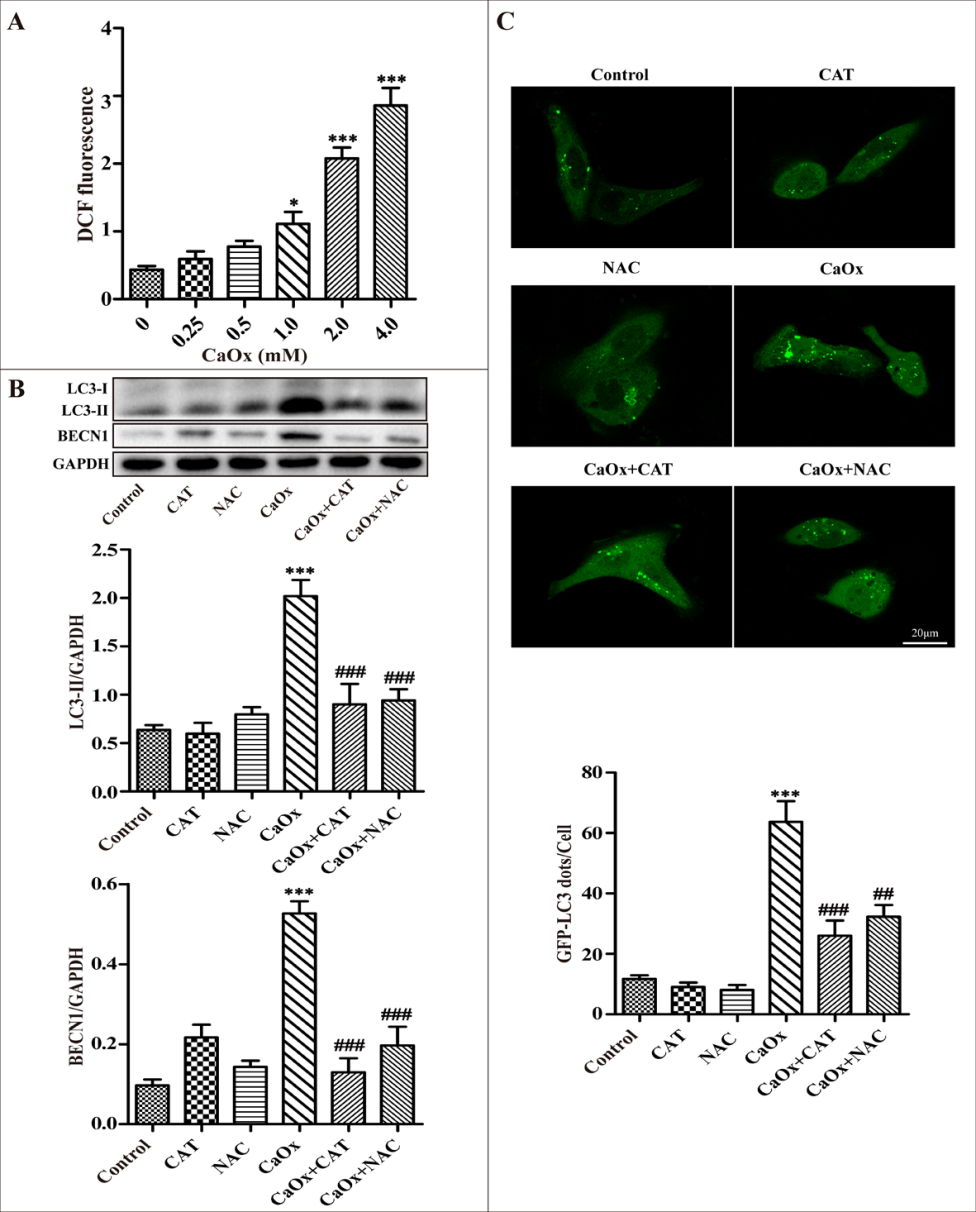
ROS mediates CaOx crystal-induced autophagy in HK-2 cells (**A**) Intracellular production of ROS was measured using a fluorescence spectrometer after HK-2 cells were treated with various concentrations of CaOx crystals. (**B**) A representative immunoblot and quantification analysis of LC3-II and BECN1 as assayed after exposure to vehicle or CaOx crystals (4 mM) in the absence or presence of catalase (CAT, 2000 U/ml) or NAC (5 mM) for 24 h. (**C**) The formation of GFP-LC3 dots was analyzed using confocal microscopy after exposure to vehicle or CaOx crystals (4 mM) in the absence or presence of catalase (CAT, 2000 U/ml) or NAC (5 mM) for 24 h. GFP-LC3 dots/cell were quantified; scale bar: 20 μm. Data are presented as the mean ± SD from three experiments. ^*^P < 0.05, ^***^P < 0.001 versus the control group, ^##^*P* < 0.01, ^###^*P* < 0.001 versus the CaOx (4 mM) group.

We tested whether CaOx crystal-induced autophagy is mediated through ROS generation in renal tubular epithelial cells. Preliminary tests demonstrated that exposure of renal tubular epithelial cells to different concentrations of CaOx crystals resulted in a dose-dependent increase in the generation of ROS (Figure 3A). Next, we observed that administration of antioxidants N-acetylcysteine (NAC) or catalase (CAT) attenuated CaOx crystal-induced accumulation of autophagosomes, whereas NAC and catalase themselves did not affect autophagosome formation (Figure 3B–3C). Taken together, these findings indicate that ROS plays an important role in CaOx crystal-induced autophagy in renal tubular epithelial cells.

### Inhibition of autophagy attenuated CaOx crystal-induced HK-2 cell injury *in vitro*

**Figure 4:**
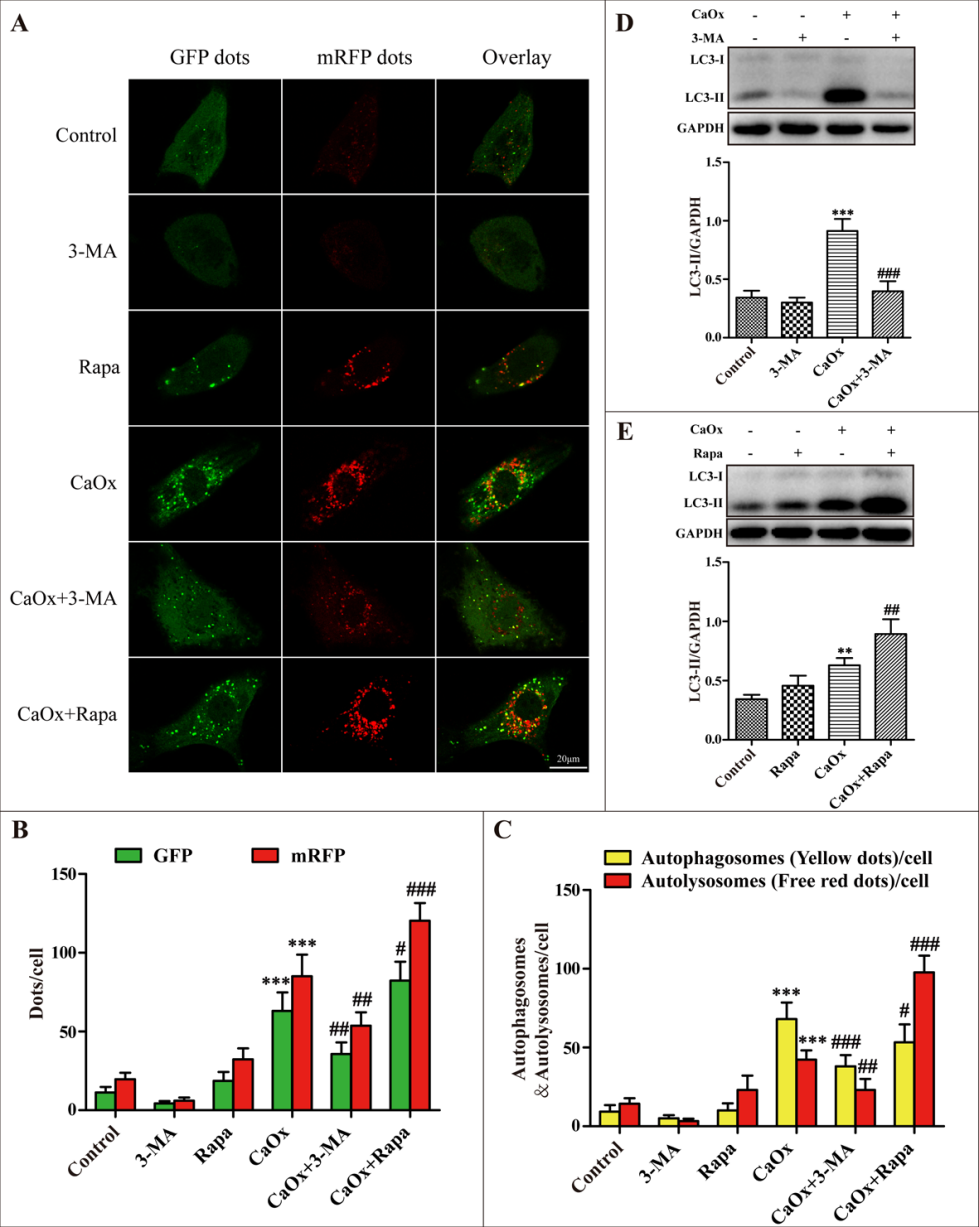
Effects of 3-methyladenine and rapamycin on CaOx crystal-induced autophagy (**A**) HK-2 cells were transduced with Ad-mRFP-GFP-LC3 and then treated with vehicle or CaOx crystals (4 mM), with or without 3-methyladenine (3-MA, 5 mM) or rapamycin (Rapa, 10 μM) for 24 h. Under confocal microscopy, GFP dots displayed as green fluorescence while mRFP dots are red. In the merged images, autophagosomes and autolysosomes are labeled with yellow and red dots, respectively; scale bar: 20 μm. (**B**) Mean numbers of GFP and mRFP dots per cell. (**C**) Mean numbers of autophagosomes and autolysosomes per cell. (**D** and **E**) Representative immunoblot and quantification analysis of LC3-II as assayed after exposure to vehicle or CaOx crystals (4 mM) in the absence or presence of 3-methyladenine (3-MA, 5 mM) or rapamycin (Rapa, 10 μM) for 24 h. Data are presented as the mean ± SD from three experiments. ^**^P < 0.01, ^***^P < 0.001 versus the control group, ^#^*P* < 0.05, ^##^*P* < 0.01 and ^###^*P* < 0.001 versus the CaOx (4 mM) group.

The autophagic inhibitor 3-methyladenine (3-MA) inhibits autophagosome formation from the beginning stage while rapamycin (Rapa) promotes fusion of the autophagosome with lysosome and thereby increases autophagic flux. We therefore investigated whether these 2 drugs were able to regulate the levels of autophagy induced by CaOx crystals. We used an adenovirus harboring tandem fluorescent mRFP-GFP-LC3 to evaluate autophagic flux in cultured HK-2 cells. Under confocal microscopy, mRFP dots were red while GFP dots were green. In the merged images, autophagosomes and autolysosomes are labeled with yellow and red dots, respectively. Compared with the control group, the numbers of green, red and yellow dots were markedly increased after treatment with CaOx crystals (Figure 4A–4C). Rapamycin pretreatment resulted in more autophagosomes and autolysosomes than in the CaOx crystal-treated group, suggesting that rapamycin promoted autophagic flux (Figure 4A–4C). However, the numbers of autophagosomes and autolysosomes significantly decreased after pretreatment with 3-MA, indicating that 3-MA impaired autophagic flux (Figure 4A–4C). As shown in Figure 4D and 4E, the expression levels of LC3-II induced by CaOx crystals were decreased by 3-MA pretreatment but enhanced by rapamycin. Together, these results suggest that 3-MA and rapamycin could effectively regulated autophagy response induced by CaOx crystals.

**Figure 5:**
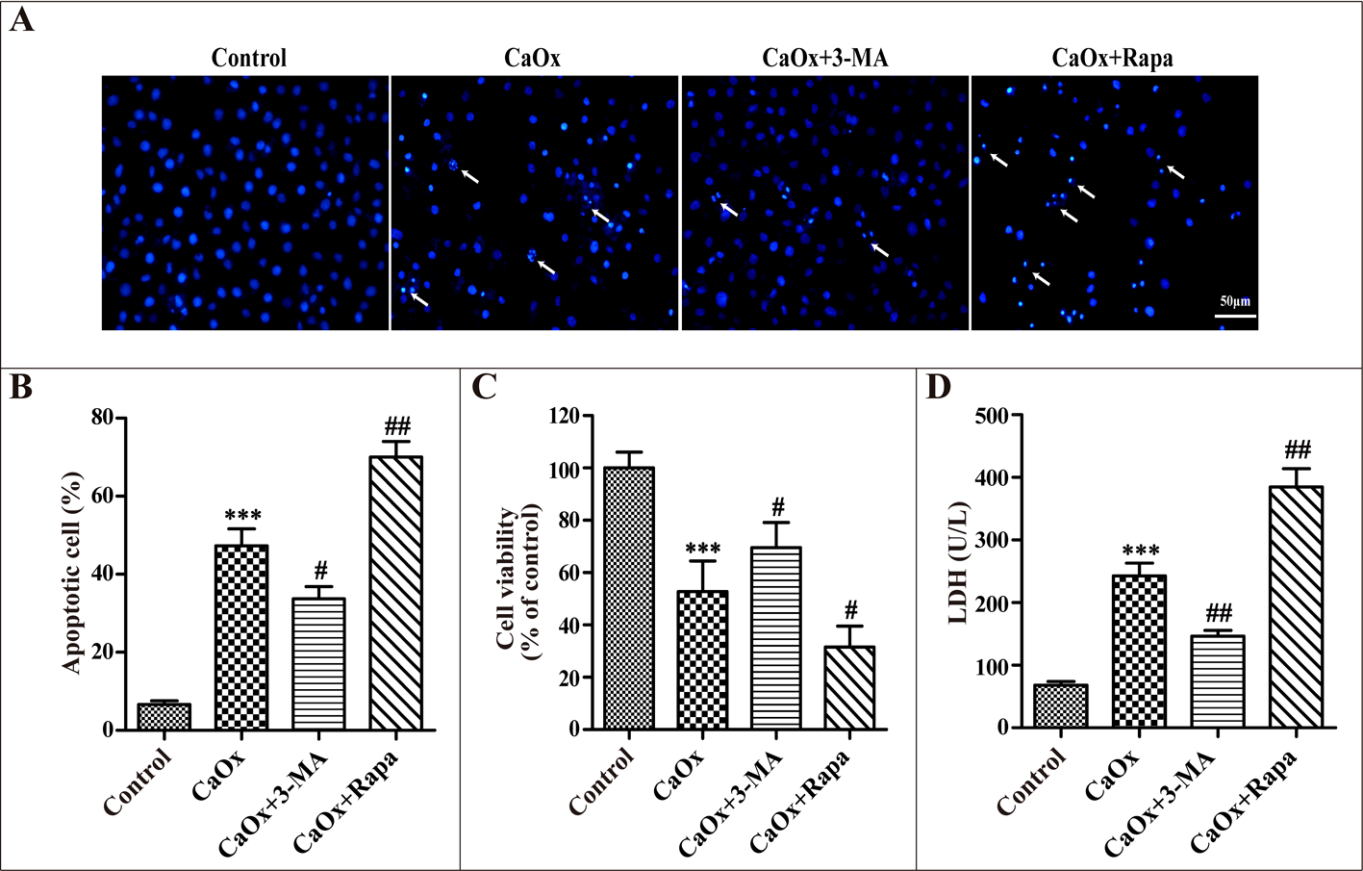
Effects of 3-methyladenine and rapamycin on CaOx crystal-induced HK-2 cell injury HK-2 cells were incubated with CaOx crystals (4 mM) for 24 h in the absence or presence of 3-methyladenine (3-MA, 5 mM) or rapamycin (Rapa, 10 μM). (**A**) DAPI staining was used to examine cell and nuclear morphology to analyze apoptosis. White arrows indicated apoptosis of HK-2 cells; scale bar: 50 μm. (**B**) Quantitative analysis of CaOx crystals induced apoptosis. (**C**) Cell viability was measured by CCK-8 assay. (**D**) The levels of LDH in culture supernatant were determined using the LDH assay. Data are presented as the mean ± SD from three experiments. ^***^P < 0.001 versus the control group, ^#^*P* < 0.05, ^##^*P* < 0.01 versus the CaOx (4 mM) group.

After confirming the effects of 3-MA and rapamycin on autophagy, we examined their effects on CaOx crystal-induced renal tubular epithelial cell injury. Compared with the control group, incubation with CaOx crystals (4 mM) for 24 h increased cellular apoptosis (Figure 5A and 5B), suppressed cell viability (Figure 5C), and enhanced lactate dehydrogenase (LDH) activity (Figure 5D). We then demonstrated that cell injury was attenuated by 3-MA but aggravated by rapamycin (Figure 5A–5D), whereas 3-MA and rapamycin themselves did not affect cell injury (data not shown).

**Figure 6:**
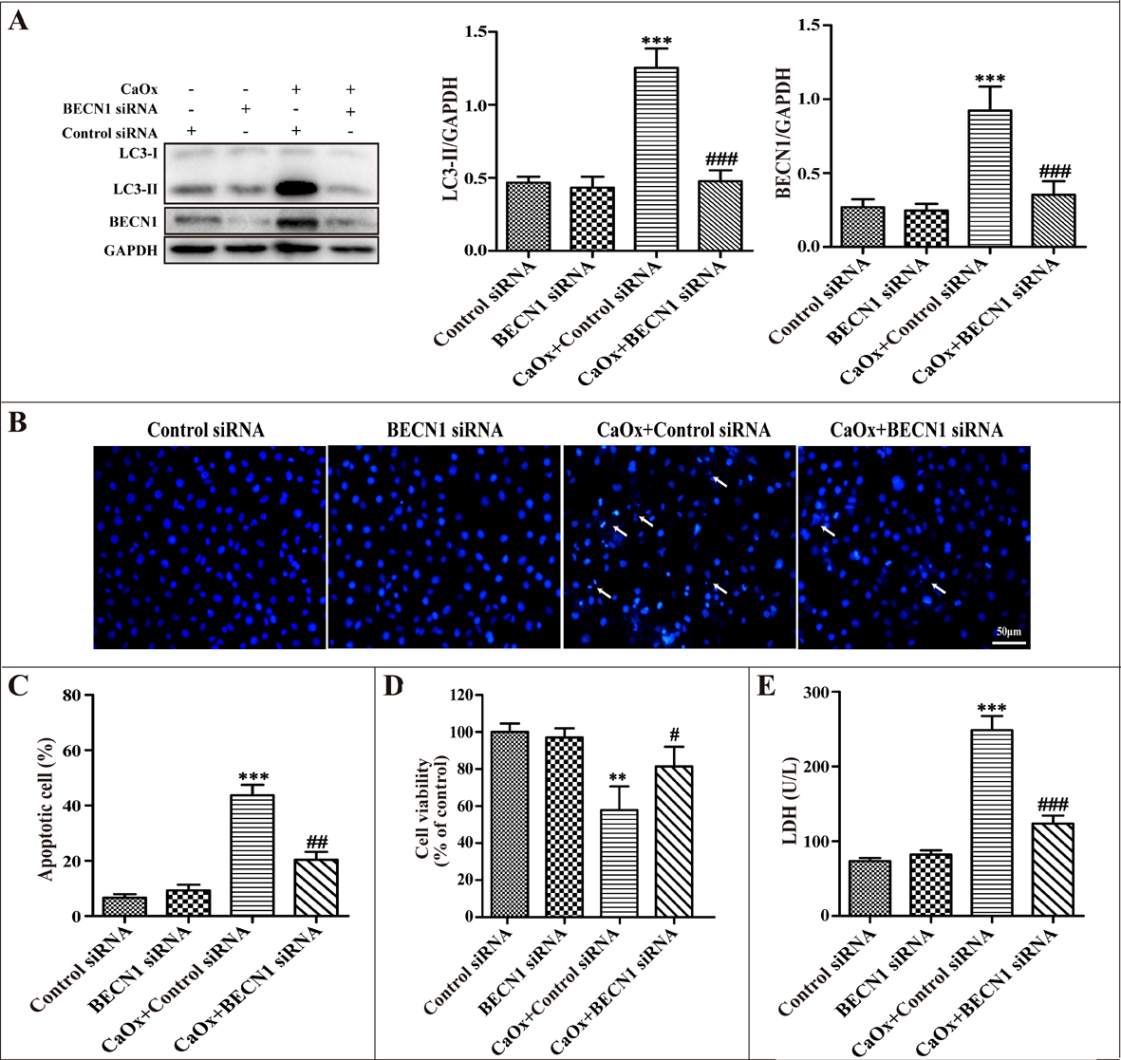
Inhibition of autophagy using siRNA knockdown of BECN1 attenuated CaOx crystal-induced cell injury HK-2 cells were transiently transfected with the negative control siRNA, BECN1 siRNA, and then incubated with CaOx crystals (4 mM) for 24 h. (**A**) Representative immunoblot and quantification analysis of LC3-II and BECN1. (**B**) Apoptosis was assessed by DAPI staining. White arrows indicated apoptosis of HK-2 cells; scale bar: 50 μm. (**C**) Quantitative analysis of CaOx crystal-induced apoptosis. (**D**) Cell viability was measured by CCK-8 assay. (**E**) The levels of LDH in culture supernatant were determined using a LDH assay. Data are presented as the mean ± SD from three experiments. ^**^P < 0.01, ^***^P < 0.001 versus the control siRNA group, ^#^*P* < 0.05, ^##^*P* < 0.01 and ^###^*P* < 0.001 versus the CaOx (4 mM)+control siRNA group.

To further confirm the role of autophagy in cell injury during CaOx crystal treatment, we used small-interfering RNA knockdown of BECN1 to inhibit autophagy. Down-regulation of BECN1 decreased BECN1 protein expression and suppressed CaOx crystal-induced LC3-II formation (Figure 6A). Compared with the negative control siRNA group, BECN1 siRNA attenuated CaOx crystal-induced cells apoptosis (Figure 6B–6C), increased cell viability (Figure 6D), and decreased LDH activity (Figure 6E), whereas BECN1 siRNA did not affect cell injury. Taken together, these findings indicated that suppression of autophagy provided a pro-survival role in CaOx crystal-induced renal tubular epithelial cell injury.

## DISCUSSION

In the present study, we clearly demonstrate that CaOx crystals lead to the induction of autophagy in renal tubular epithelial cells both *in vivo* and *in vitro*. The results of clinical experimental studies show that CaOx crystals induced autophagy as evidenced by the elevated levels of critical autophagic proteins LC3-II and BECN1. Transmission electron microscopy, the gold standard to monitor the formation of autophagosomes, showed that autophagic vacuoles are increased in kidney tissue samples from patients with calcium oxalate nephrolithiasis. In addition, our results were partially consistent with Liu *et al.* studies showing that the dynamic process of typical initial autophagic vacuoles and late/degradative autophagic vacuoles [14]. Intriguingly, a growing body of literature suggests that LC3 immunohistochemical staining could be used to evaluate autophagy in clinical specimens [19, 20]. Our studies observed significantly accumulated LC3 and BECN1 by immunohistochemistry staining in calcium oxalate nephrolithiasis samples. Tissue culture studies show that CaOx crystals induced accumulation of autophagosomes in a dose- and time-dependent manner, as indicated by the number of GFP-LC3 dots, immunoblot analysis of BECN1, LC3-II expression, and autophagic vacuoles detected by TEM. Taken together, these data provide compelling evidence, for the first time, on the occurrence of autophagy in RTECs in response to CaOx crystals.

The pathophysiological roles of autophagy in renal tubular cell damage in various kidney diseases remain either cytoprotective or deleterious, most likely depending on the different stress factors [21–24]. Although the effect of autophagy in some renal diseases has been investigated, very little is known about the role of autophagy in the development of calcium oxalate nephrolithiasis. In the present study, we have clearly observed that autophagy was deleterious in CaOx crystal-induced renal tubular epithelial cell injury. Inhibiting autophagy using autophagy inhibitor 3-MA and small-interfering RNA to knock down of BECN1 attenuated CaOx crystal-induced RTEC injury. However, we found that pretreatment with rapamycin, an effective autophagy activator, significantly exacerbated cell injury. Therefore, these results clearly demonstrate that autophagy activation further aggravates CaOx crystal-induced RTEC injury.

Reactive oxygen species (ROS) is a general term for free radicals, atoms or molecules with unpaired electrons and their metabolites, which mainly include superoxide anion (O_2_^−^), hydrogen peroxide (H_2_O_2_), nitric oxide radical (NO.), and hydroxyl radical (OH.) [25, 26]. Under physiologic conditions, low concentrations of ROS contribute to cell proliferation, differentiation and migration [27]. In pathological states, intracellular antioxidants cannot effectively degrade ROS, leading to the development of oxidative stress (OS) that is injurious to cellular components, such as lipids, nucleic acids, proteins, and DNA, ultimately causing serious cell and tissue injury [28, 29]. Therefore, intracellular ROS production plays a key regulatory role in some disease pathophysiological processes.

Our current results demonstrate that intracellular ROS generation may participate in CaOx crystal-induced autophagy. First, we found that exposure of renal tubular epithelial cells to different concentrations of CaOx crystals resulted in a dose-dependent increase in the generation of ROS. Next, our data suggest that administration of antioxidants N-acetylcysteine (NAC) or catalase significantly attenuated CaOx crystal-induced accumulation of autophagosomes, whereas NAC and catalase themselves did not affect autophagosome formation.

Previous studies have shown that treatments with antioxidants (NAC, catalase) and free radical scavengers significantly reduced the production of ROS and decreased Ox/CaOx crystal-induced renal injury and inflammation [30–32]. Based on these findings, we inferred that in the internal environment of calcium oxalate renal calculi, overproduction of ROS mediated autophagy may up-regulate the release of inflammatory factors and increase the apoptosis and necrosis of the renal tubular epithelial cells, ultimately promoting calcium oxalate stone formation.

Mitochondria are major intracellular sources of ROS, and mitochondrial damage is accompanied by the production of ROS [33]. In the calcium oxalate nephrolithiasis patients, observations by TEM showed that mitochondria were swollen and damaged. A previous study revealed that calcium oxalate monohydrate increased mitochondria $O_{2}^{-}$ production in renal epithelial cells [34]. In addition, mounting evidence shows that mitochondria have an essential role in regulating autophagy [35–37]. Therefore, we assume that mitochondrial damage may exert a crucial effect in CaOx crystal-induced autophagy.

Both 3-MA and chloroquine (CQ) could effectively inhibit autophagy through various mechanisms. In *in vitro* experiments, we demonstrated that CQ also could effectively inhibit CaOx crystal-induced autophagy and attenuated renal tubular epithelial cell injury (data not shown). In addition, our results suggest that inhibition of autophagy by knockdown of BECN1 significantly attenuated CaOx crystal-induced RTEC injury and apoptosis, compared with autophagy inhibitor 3-MA. We inferred that gene-knockdown approaches may be able to more effectively inhibit autophagy activation.

In conclusion, our results demonstrated for the first time that autophagy induced by CaOx crystals via the ROS pathway exacerbated renal tubular epithelial cell injury. Inhibiting autophagy could be a novel therapeutic strategy for CaOx nephrolithiasis. However, the more detailed mechanisms of ROS-mediated autophagy in CaOx crystal-induced renal tubular epithelial cell injury still need to be clarified *in vivo* and *in vitro*.

## MATERIALS AND METHODS

### Reagents and antibodies

Calcium oxalate (Sigma, 455997), 3-methyladenine (Sigma, M9281), rapamycin (Sigma, R0395), 4′,6-diamidino-2-phenylindole (Sigma, D8417), N-acetyl-L-cysteine (Sigma, A7250), catalase (Millipore, 219261-100KU), 2′,7′-dichlorofluorescin diacetate (Sigma, D6883), Lipofectamine 3000 (Invitrogen, L3000008), and rabbit anti-LC3B (Sigma, L7543) for western blot (WB) (1:2000), mouse anti-BECN1 (Cell Signaling Technology, 3495) for WB (1:1000). Rabbit anti-LC3B (Abcam, ab51520) (1:1000) and mouse anti-BECN1 (Abcam, ab114071) were used for immunohistochemistry (IHC) (1:500). Mouse monoclonal anti-GAPDH (Proteintech, 60004-1-Ig) was used for WB (1:5000). Mouse and rabbit HRP-conjugated antibodies were obtained from Zhongshan Golden Bridge Biotechnology (ZB-2305, ZB- 2301).

### Patient material

Ethical approval of this study was granted by the Ethical Review Committee of the First Affiliated Hospital of Guangxi Medical University. In total, 28 kidney tissue specimens were collected from patients with calcium oxalate nephrolithiasis (the main component is calcium oxalate by stone composition analysis). A total of 12 normal kidney tissue samples, which were confirmed by histopathological analysis, were obtained from patients who underwent radical nephrectomy of tumors as a control group.

### Cell culture studies

Human proximal tubular HK-2 cells (American Type Culture Collection) were maintained in DMEM/F12 supplemented with 10% FBS, 100 U/ml penicillin/streptomycin at 37°C under 5% CO_2_. HK-2 cells were exposed to 0, 0.1, 0.25, 0.5, 1, 2, and 4 mM CaOx crystals for 0, 2, 4, 8, 12 and 24 h before analysis. Then, the protein levels of LC3-II and BECN1 were measured by western blot. Subsequently, the cells were treated with rapamycin (10 μM), 3-MA (5 mM), NAC (5 mM), and catalase (2000 U/ml) with or without CaOx crystals (4 mM) for 24 h. The levels of lactate dehydrogenase (LDH) in culture supernatant were determined using a lactate dehydrogenase (LDH) assay kit.

### Histology and immunohistochemical staining

The kidney specimens from human were fixed with 4% paraformaldehyde and embedded in paraffin. Tissue sections (4 mm) were stained with hematoxylin and eosin (H&E) to assess histological tissue injury. For immunohistochemical staining, sections were deparaffinized and rehydrated. Sections then underwent antigen retrieval with 0.01 mol/L sodium citrate, followed by blocking with 5% bovine serum albumin (BSA) and incubation with primary antibody at 4°C overnight. Sections were incubated with streptavidin-HRP at room temperature for 30 min, then stained with 3,3-diaminobenzidine (DAB) substrate. Finally, sections were counterstained with hematoxylin and images were acquired under a microscope (Olympus C-5050, Japan).

### Western blot analysis

In brief, proteins from tissue samples or cells were obtained using RIPA lysis buffer (Beyotime, P0013B), supplemented with 1 mM PMSF (Beyotime, ST506). Lysate protein concentration was measured by BCA protein assay kit (Beyotime, P0012). Equal amounts of protein samples were separated by 12% SDS-PAGE, and the protein bands were transferred onto PVDF membranes (Millipore, USA). After blocking with 5% skim milk for 1 h at room temperature (RT), the membranes were incubated with primary antibody overnight at 4°C, followed by the appropriate horseradish peroxidase-conjugated anti-mouse/rabbit IgG (Zhongshan Golden Bridge Biotechnology, ZB-2305/ZB-2301). Chemiluminescent signals were captured by a CCD camera in a chemiDoc XRS (Bio-Rad) instrument with Image Lab software.

### ROS detection

The intracellular ROS generation was measured using 2′,7′-dichlorofluorescin diacetate (DCFDA). After treatment with or without different concentrations of CaOx crystals for 24 h, the cells were incubated with DCFDA at 1:1000 dilution in the serum-free culture medium and maintained at 37°C for 30 min, then washed with PBS and the fluorescent intensity was measured by a spectrofluorometer (Molecular Devices, United Kingdom) with excitation and emission wavelengths of 485 and 528 nm, respectively.

### Transmission electron microscopy

Fresh kidney tissues and HK-2 cells were fixed with 2.5% glutaraldehyde in 0.1 M PBS (pH 7.4), followed by treatment with 1% osmium tetroxide, then dehydrated and infiltrated with epoxy resin. Ultrathin sections were stained with uranyl acetate and lead citrate, and these samples were subsequently observed using a Hitachi-7650 transmission electron microscope (Hitachi Instrument, Tokyo, Japan). Quantification of autophagic vesicles was performed as described previously [38, 39].

### Cell viability and apoptosis assay

Cell viability was determined using Cell Counting Kit-8 (CCK-8, Dojindo, Kumamoto, Japan) according to the manufacturer's protocol. Briefly, approximately 1 × 10^4^ cells were seeded in 96-well plates and cultured for 24 h at 37°C in a CO_2_ incubator. After being treated, 100 μl of fresh medium and 10 μl of CCK-8 solution were added to each well. The plates were then incubated for 2 h at 37°C. Subsequently, the optical density was measured using a microplate reader (Bio-Tek, USA) with the absorbance at 450 nm. To assess apoptosis, cells were stained with 4′6-diamidino-2-phenylindole (DAPI) at the end of the experiment. Cellular and nuclear morphology were then observed under fluorescence microscopy. For cell count, four fields with ∼200 cells per field were evaluated in each dish to estimate the percentage of apoptosis.

### Adenovirus infection

To evaluate autophagic flux in cultured HK-2 cells, we used an adenovirus harboring tandem fluorescent mRFP-GFP-LC3. The mRFP-GFP-LC3 adenoviral vectors were obtained from Hanbio, Inc. (Shanghai, China). Briefly, cells were infected with the tandem fluorescent mRFP-GFP-LC3 adenoviral vectors for 24 h, and then subjected to further study. The autophagic flux was determined by evaluating the number of GFP and mRFP dots under confocal microscopy as described previously [40].

### Plasmids, siRNAs, and transfections

HK-2 cells were transiently transfected with GFP-LC3 plasmid or small interfering RNA (siRNA) against BECN1 using Lipofectamine 3000 (Invitrogen, L3000015) according to the manufacturer's instructions. The GFP-LC3 (24920) plasmid was purchased from Addgene. The negative control and BECN1 siRNA were purchased from Santa Cruz Biotechnology. After transfection, the cells were treated according to experimental needs. The numbers of GFP-LC3 dots were quantified from at least 30 cells per sample using confocal microscopy. Knockdown efficiencies or specific gene expression were determined by western blot analysis.

### Statistical analysis

All of the data were analyzed using SPSS 20.0 software. Data are presented as the mean ± SD from three experiments. Significant differences between two groups were determined by Student's *t*-test. Significant differences in multiple groups were analyzed with one-way analysis of variance (ANOVA). *P* < 0.05 was considered statistically significant.
